# Clinical Pharmacology and Approach to Dose Selection of Emestedastat, a Novel Tissue Cortisol Synthesis Inhibitor for the Treatment of Central Nervous System Disease

**DOI:** 10.1002/cpdd.1496

**Published:** 2025-01-02

**Authors:** Paul Rolan, Jonathan Seckl, Jack Taylor, John Harrison, Paul Maruff, Michael Woodward, Richard Mills, Mark Jaros, Dana Hilt

**Affiliations:** ^1^ Actinogen Medical Ltd Sydney New South Wales Australia; ^2^ University of Adelaide Medical School Adelaide South Australia Australia; ^3^ Queen's Medical Research Institute University of Edinburgh Edinburgh UK; ^4^ Scottish Brain Sciences Edinburgh UK; ^5^ King's College London UK; ^6^ Alzheimercentrum, Amsterdam University Medical Center Amsterdam The Netherlands; ^7^ Florey Institute for Neuroscience and Mental Health Parkville Victoria Australia; ^8^ Medical Health and Cognitive Research Unit, Austin Health Heidelberg Repatriation Hospital Heidelberg West Victoria Australia; ^9^ Icon Clinical Research Inc Reading UK; ^10^ Summit Analytical LLC Denver CO USA

**Keywords:** 11β‐HSD1, Alzheimer disease, cognitive enhancement, cognitive test battery, cortisol, dose‐finding, PET

## Abstract

This review demonstrates the value of central pharmacodynamics (PD), including positron emission tomography (PET) and computerized cognitive testing, to supplement pharmacokinetic (PK) and peripheral PD for determining the target dose range for clinical efficacy testing of emestedastat, an 11β‐hydroxysteroid dehydrogenase 1 (11β‐HSD1) inhibitor. Combined data from 6 clinical trials in cognitively normal volunteers and patients with Alzheimer disease included a population PK model, endocrine PD, a human PET trial (11β‐HSD1 brain imaging), and computerized cognitive testing. PK and PET findings were similar in volunteers and patients with Alzheimer disease. PK modeling suggested that 20 mg daily would be optimal to maintain cerebrospinal fluid concentrations above the brain half maximal inhibitory concentration. However, subsequent PET scanning suggested that emestedastat doses of 10 or even 5 mg daily may be sufficient to adequately inhibit 11β‐HSD1. With once‐daily doses of 5‐20 mg in cognitively normal, older volunteers, a consistent pattern of pro‐cognitive benefit, without dose‐response, was seen as improvement in attention and working memory but not episodic memory. Thus, emestedastat therapeutic activity might be attained at doses lower than those predicted from cerebrospinal fluid drug levels. Doses as low as 5 mg daily may be efficacious and were studied in subsequent trials.

Selecting the optimal dose for further clinical development is especially problematic for drugs directed at the central nervous system (CNS), especially if the target is differentially expressed in regions of the CNS. Cerebrospinal fluid (CSF) drug levels may correlate with CNS concentrations but are not always representative of brain tissue maximum concentration levels, especially if single‐point sampling is used, as is often the case for pragmatic reasons. For drugs with a novel mechanism of action, these problems are often greater.

Emestedastat (provisional international nonproprietary name), previously designated UE2343 and Xanamem, is an orally active small molecule discovered at the University of Edinburgh that inhibits the tissue cortisol synthesis enzyme, 11β‐HSD1 but not the principal cortisol synthesis pathway in the adrenal glands via 17‐hydroxylase (Figure 1).^1^, ^2^

**Figure 1:**
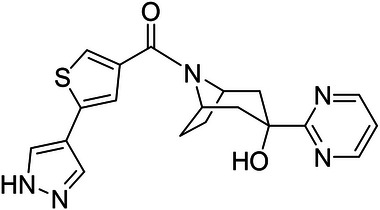
Chemical structure of emestedastat [(5‐(1H‐Pyrazol‐4‐yl)thiophen‐3‐yl)(3‐hydroxy‐3‐(pyrimidin‐2‐yl)‐8 azabicyclo[3.2.1]octan‐8‐yl) methanone].

11β‐HSD1 catalyzes the intracellular synthesis of the active glucocorticoid cortisol from inert cortisone in humans. 11β‐HSD1 is widely distributed, with the highest expression in the liver. The enzyme also is expressed at high levels in adipose tissue, skeletal and vascular smooth muscle, brain (neurons and glia), and leukocytes.^3^ In the brain, 11β‐HSD1 is highly expressed in the hippocampus, cortex, and cerebellum, regions essential for cognition. In particular, the hippocampus highly expresses both types of corticosteroid receptors (type I, or mineralocorticoid receptors; and type II, or glucocorticoid receptors) and is a key locus for glucocorticoid actions on learning and memory.^3^, ^4^ Emestedastat is in clinical development for psychiatric and neurological diseases in which excess intracerebral cortisol activity is thought to contribute to pathogenesis.

The evidence for the toxicity of excess cortisol in the brain comes from multiple sources. Age increases 11β‐HSD1 activity in the hippocampus of male macaques, possibly increasing cortisol neurotoxicity.^5^ Chronically elevated circulating cortisol levels, as observed in Cushing disease, severe depression, or with corticosteroid pharmacotherapy, are associated with numerous deleterious metabolic and neuropsychiatric consequences including cognitive dysfunction,^6^ depression,^7^, ^8^, ^9^ and neurotoxicity.^10^ Elevated plasma and CSF cortisol levels are strongly associated with Alzheimer disease (AD).^11^, ^12^

11β‐HSD1 acts as an intracellular amplifier of glucocorticoid action in target tissues where it is expressed, for instance, contributing approximately 50% of intrahippocampal corticosteroid levels.^3^ Animal and human studies have shown that specific inhibition of 11β‐HSD1 reduces intracellular cortisol levels and may provide a therapeutic strategy to treat cardiometabolic, neurodegenerative, and neuropsychiatric diseases characterized by persistently elevated cortisol levels.^13^, ^14^, ^15^, ^16^, ^17^, ^18^, ^19^ These findings underpin the development of emestedastat for AD and other cognitive impairments associated with age or disease. Animal studies suggest that only moderate inhibition of 11β‐HSD1 is necessary for enzyme inhibition and therapeutic effect. A study in mice with age‐associated cognitive impairment demonstrated that partial inhibition (27% inhibition in the hippocampus) of brain 11β‐HSD1 using the inhibitor UE1961 was sufficient to produce an improvement in spatial memory.^13^ This effect was also observed in transgenic (*Hsd11b1^−/+^*) mice displaying partial knockdown of endogenous 11β‐HSD1 levels (translating to approximately 40% of normal residual enzyme activity), suggesting that substantial improvements in memory can be achieved with submaximal levels of brain inhibition. Another study in aged mice and a murine Tg2576 model of AD was conducted using the inhibitor UE2316,^17^ which produced approximately 30% inhibition of 11β‐HSD1 in the brain, accompanied by improved memory and reduction in amyloid‐β in the cortex.

Taken together, these studies provide evidence that partial inhibition of 11β‐HSD1 in the brain may be sufficient to produce cognitive enhancement in the context of aging and AD. If translated to humans, inhibition of brain 11β‐HSD1 greater than 30% would be sufficient to provide cognitive enhancement. This set a target for the clinical pharmacology program to identify a dosage regimen to achieve and exceed this degree of 11β‐HSD1 inhibition in the brain (60%) and to seek supportive biomarker evidence that this is likely to provide a therapeutically effective exposure.

One condition being explored in emestedastat development is major depressive disorder. For decades, the evidence has suggested that dysregulation of the hypothalamic‐pituitary‐adrenal (HPA) axis is involved in the pathogenesis of depression, especially melancholic depression.^20^, ^21^, ^22^ While there have been unsuccessful attempts to develop compounds specifically targeted to this mechanism, such as the corticotropin‐releasing hormone inhibitors,^23^ a meta‐analysis of clinical trials of cortisol synthesis inhibitors in depression concluded that inhibiting this target was likely to be efficacious.^24^ Although these trials were useful as a proof of principle, compounds targeting systemic cortisol, such as the cortisol synthesis inhibitor metyrapone and the nonselective mifepristone, were not suitable for development as treatments for this condition due to significant adverse peripheral hormonal effects.

In this article, we describe the clinical pharmacology and approach to dose finding for emestedastat, including the 4 complementary approaches used to select its target dose range for Phase 2 and Phase 3 trials.

## Methods

### Completed Clinical Trials

The clinical pharmacology trials and clinical trials completed to date, including key design features, are listed in Table 1. All trials were approved by a local ethics committee/institutional review board, and all participants gave written informed consent. Details of the trial sites and institutional review boards are presented in Table S1. Endocrinology data and a population PK (PPK) analysis are available from the following trials:

· A single‐ascending‐dose (SAD) trial in healthy volunteers (RD 656/25368)^25^
· A multiple‐ascending‐dose (MAD) trial in healthy volunteers, including a small subtrial measuring CSF concentrations (ACW0001)^25^
· A positron emission tomography (PET) trial in cognitively normal, older volunteers and patients with AD (ACW0004)^26^
· Two trials in cognitively normal, older volunteers examining safety and effects on cognition (Trials ACW0003, NCT03830762 and ACW0005; and NCT04983368)
· A PPK analysis from the healthy volunteer and patient trials
· HPA axis endocrinology data from the volunteer and patient trials
· A trial of efficacy and safety in patients with AD (ACW0002; NCT02727699)^27^

**Table 1:** Summary of Clinical Trials With Emestedastat

Trial identifier (NCT reference)	Trial design	Participants (N)	Oral dosing regimen (daily dose)	Dosing duration
RD 656/25368^25^	DB, R, PC, SAD	Healthy volunteers (48)	0, 2, 5, 10, 18, 25, and 35 mg	Single dose
ACW0001^25^	DB, R, PC, MAD	Healthy volunteers (24)	0, 20, 40, and 70 mg	Daily dosing split twice daily (12‐hourly) for 9 days (Days 1‐9) and once on Day 10 (19 doses in total)
	Fed–fasted PK subtrial OL, R, CO	Healthy volunteers (12)	35 mg	Single‐dose crossover
	OL, NR CSF/plasma PK subtrial	Healthy volunteers (4)	35 mg	Twice daily (12‐hourly) for 3 days (Days 1‐3) and once on Day 4 (7 doses in total)
XanaHES ACW0003 (NCT03830762)^a^	SB (central reader), R, PC	Cognitively normal, older volunteers (42)	0, 20 mg	Once‐daily for 12 weeks
ACW0004^26^	PET trial OL, NR	Cognitively normal, older volunteers/patients with MCI/AD (40)	5, 10, 20, and 30 mg	Once daily for 7 days
XanADu ACW0002 (NCT02727699)^b^	DB, R, PC, central reader‐blinded	Patients with AD (185)	0, 10 mg	Once daily for 12 weeks
XanADu biomarker extension ACW0002A^27^	DB, R, PC, central reader‐blinded	Patients with AD and stored plasma from ACW0002 (72)	0, 10 mg	Once daily for 12 weeks
XanaMIA‐DR ACW0005 (NCT04983368)^c^	DB, R, PC	Cognitively normal, older volunteers (107)	0, 5, and 10 mg	Once daily for 6 weeks

AD, Alzheimer disease; CO, crossover; CSF, cerebrospinal fluid; DB, double‐blind; MAD, multiple‐ascending‐dose; MCI, mild cognitive impairment; NCT, National Clinical Trial; NR, not randomized; OL, open‐label; PC, placebo‐controlled; PK, pharmacokinetics; R, randomized; SAD, single ascending dose; SB, single‐blind.

a https://clinicaltrials.gov/study/NCT03830762

b https://clinicaltrials.gov/study/NCT02727699

c https://clinicaltrials.gov/study/NCT04983368

Initially, the SAD and MAD trials studied doses up to 35 mg twice daily and assessed PK and HPA PD.

The next trial (ACW0002/XanADu; clinical trials.gov NCT02727699) was largely designed pragmatically, based on the SAD and MAD data that included 4 individuals with single‐point CSF sampling and 13‐week animal toxicology studies. It was a placebo‐controlled, double‐blind, Phase 2a trial of 12 weeks’ duration using 10 mg daily in 185 patients with a clinical diagnosis of mild AD without biomarker or PET scan confirmation of amyloid‐based disease. The primary efficacy end points were the AD Assessment Scales—Cognitive Subscale Version 14 (ADAS‐Cog v14) and the AD COMposite Scores (comprising weighted composite data derived from the ADAS‐Cog v14, Clinical Dementia Ratio—Sum of Boxes, and Mini‐Mental State Examination). Sparse PK and HPA PD sampling were also measured. Subsequently, a new in silico trial (ACW0002A) was performed using available stored plasma from the original trial to evaluate efficacy in those participants where elevated plasma pTau181 level confirmed a diagnosis of AD.^27^

The next trial, XanaHES (ACW0003) was a double‐blind, placebo‐controlled, Phase 1b trial in cognitively normal, older participants (age range, 50‐75 years) at a dose of 20 mg daily for 12 weeks. The population of 42 participants comprised 30 receiving emestedastat and 12 receiving placebo. The objectives were to assess safety, PK, HPA PD, and cognition. The computerized cognitive test battery (CTB; Cogstate) assessed attention, working memory, and episodic memory.

XanaMIA‐DR (ACW0005) was a double‐blind placebo‐controlled Phase 1b trial in 107 healthy older participants (age range, 50‐80 years) at doses of 5 and 10 mg daily for 6 weeks. The principal objective of XanaMIA‐DR was to assess whether the improvements in cognition observed in XanaHES were also present at the lower doses of 5 and 10 mg daily.

Details of the analytical methods used for the determination of plasma levels of emestedastat and cortisol, testosterone, androstenedione, dehydroepiandrosterone sulfate (DHEAS), and adrenocorticotropic hormone (ACTH) are presented in the Supplemental Information.

### Positron Emission Tomography Imaging of Cerebral 11β‐HSD1 Occupancy

In parallel with the XanaHES trial, a PET Phase 1 trial was performed using an 11β‐HSD1‐specific radiolabeled tracer (^11^C‐TARACT, a specific imaging ligand competitive with emestedastat for binding to 11β‐HSD1) to compare target enzyme binding/occupancy in the brain at multiple doses of 5, 10, 20, and 30 mg daily for 7 days in both cognitively normal, older participants (n = 23) and patients with AD (n = 17).^26^ This included both morning and evening PET scans that were performed at 4‐7 or 16‐18 hours after dosing. There were 3 or 4 participants per group.

### Cognition

In both XanaHES and XanaMIA‐DR, cognition was assessed using a computerized CTB (Cogstate). The CTB comprised tests indexing key domains of cognition including processing speed, attention, visual learning, working memory, psychomotor function, and executive function. In XanaHES, least squares mean differences between the emestedastat 20 mg treatment group and the placebo group were estimated and tested at a 2‐sided 0.05 significance level. No adjustments for α were made for multiple comparisons. In XanaMIA‐DR, standardized effect sizes, equivalent to a Z score, were calculated with a threshold for clinically meaningful effect set at 0.9, representing a 1‐sided 80% confidence interval (CI).

The individual tests from the Cogstate battery were combined into 2 composite scores indexing:

· “Attention Composite” of attention and working memory (Detection Test, Identification Test, One Back Test)
· “Memory Composite” of episodic memory and learning (One Card Learning Test, Continuous Paired Associate Learning Test, and Continuous Paired Associate Learning Test‐Delayed Recall).

To compute the Attention and Memory Composite scores, each of the component‐test scores were Z transformed, the directionality of scores was reversed so that higher scores indicate better performance, and the component Z scores were averaged.

### Population Pharmacokinetics

PPK analysis was performed on data from 3 trials: the MAD trial in volunteers; the XanADu trial in patients with AD; and XanaHES in older, cognitively normal participants. In total, 138 participants (18 healthy volunteers, 90 patients with AD, and 30 healthy older participants) contributed a total of 1087 evaluable plasma concentration observations.

The analyses were performed using NONMEM Version 7.3.0 (ICON plc). PK parameter relationships were sought with the covariates body weight, age, sex, and healthy versus AD. The selected PPK model was a one‐compartment model with first‐order absorption and elimination. It was successfully fitted using Monte Carlo Importance Sampling Expectation Maximization estimation and included separate relative bioavailability terms for the Day 1 PK data from the XanADu and XanaHES trials. The M3 method was used to permit the inclusion of the below‐the‐limit quantification concentrations in the analysis. The model estimated between‐subject variation in all 4 parameters and used a full ω block to estimate the covariance between parameters. The model included a weight effect on the apparent volume of distribution.

### Pharmacodynamics—HPA Axis Endocrinology

The effects of emestedastat on plasma or serum levels of ACTH, cortisol, DHEAS, androstenedione, and testosterone in the SAD and MAD trials have been reported.^25^ The effect of emestedastat on these HPA axis hormones was further explored in the XanADu and XanaHES trials.

## Results

### Data From the SAD and MAD Trials

The SAD and MAD trials of up to 35 mg twice daily showed that a maximum tolerated dose had not been established and that emestedastat was well tolerated, with no pattern of adverse events identified. After oral administration, peak plasma drug concentrations occur after 4‐6 hours and decline, with a terminal half‐life of 10‐14 hours. There is negligible (< 5%) urinary excretion of unchanged drug, but metabolic pathways are not yet fully elucidated. CSF penetration was confirmed in 4 participants at a single time point, with a CSF : free plasma ratio of 0.33, estimated to produce concentrations adequately exceeding the half maximal inhibitory concentration for enzyme inhibition in the brain at doses from 10 mg twice daily and above.^25^ Near complete inhibition of peripheral 11β‐HSD1 at all doses in the MAD trial was demonstrated by profound suppression of urinary tetrahydrocortisol/tetrahydrocortisone ratios. There was no dose‐response in hormonal PD in the MAD trial from 10 to 35 mg twice daily. PD findings from the XanADu Phase 2a trial of 10 mg daily were similar to those for 10 mg twice daily.

### PET Imaging of Target Occupancy in the Brain

The PET trial showed that displacement of the tracer was high in all areas of the cortex (generally greater than 60%‐80% with morning dosing). Similar values for cognitively normal patients and patients with AD were observed. Occupancy in the cerebellum was lower (by about half) at 5 mg and less so at higher doses (Figure S1). With an evening dose of 10 mg, occupancy was lower in most regions by about 25% compared with morning dosing, except for the cerebellum, where it was 75% less.

The PET trial suggested that morning dosing with 10 mg should be sufficient to have full clinical effect and raised the possibility that 5 mg could be clinically active.

### Population Pharmacokinetics

PPK parameter estimates for the final PPK model are listed in Table 2.

**Table 2:** PPK Parameter Estimates for the Final PPK Model

Model parameters (units)	Estimate	%RSE	Lower 95% CI	Upper 95% CI	Between‐participant variability (CV%)	Shrinkage (%)
Structural parameters						
CL/F (L/h)	4.76	9.73	3.85	5.67	50.2	3.77
V/F (L)	64.3	4.70	58.4	70.2	33.0	12.8
k_a_ (hour^−1^)	1.74	92.5	−1.42	4.90	51.5	32.1
ALAG (hour)	0.900	38.1	0.228	1.57	75.8	22.2
Covariates						
WT on V/F	1.00	‐	‐	‐		

Half‐life was not estimated in the model, but from k_el_ = CL/V and t_1/2_ = ln(2)/K_el_ the mean elimination half‐life (95% CI) is 9.36 (8.58‐10.51) hours.

ALAG, absorption lag time; CI, confidence interval; CL/F, apparent clearance; CV, coefficient of variation; F, bioavailability; k_a_, absorption rate; PPK, population pharmacokinetic; RSE, relative standard error; V/F, apparent volume of distribution; WT, weight.

Key findings from the PPK analysis were:

· The PK of emestedastat is consistent between healthy volunteers, participants with AD, and healthy older participants.
· The multiple‐dose PK of emestedastat were linear across the dose range evaluated.
· The only covariate relationship found was between volume of distribution and body weight.

There is no need for dose adjustment due to age or body weight on the basis of PK.

**Figure 2:**
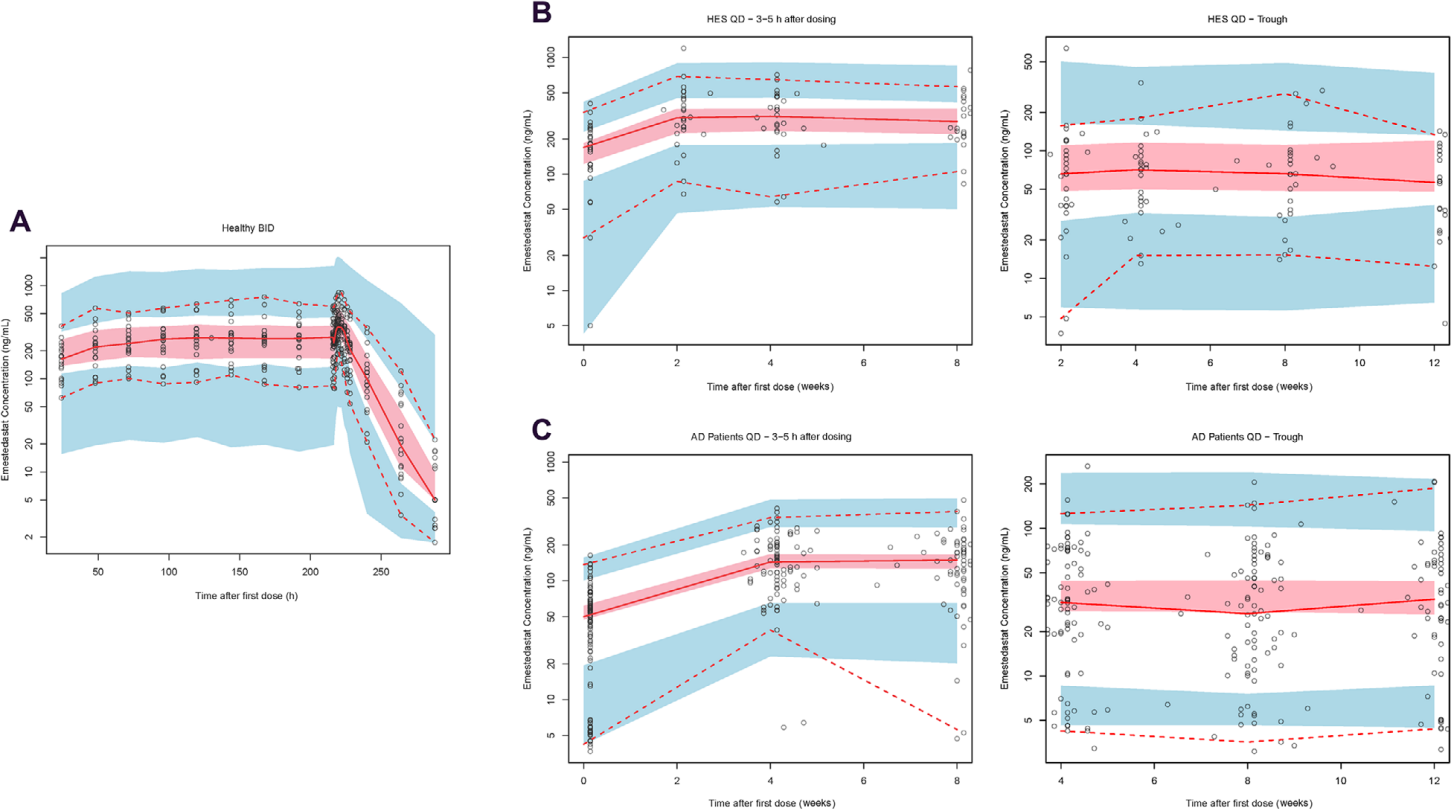
Composite VPC plots from the population PK analysis, showing postdose emestedastat plasma concentrations. (A) VPC plot for healthy participants in the MAD/ACW0001 trial. (B) VPC plots for healthy older participants in the XanaHES/ACW0003 trial, taken 3‐5 hours after dosing and at the trough. (C) VPC plots for participants with AD in the XanaMIA‐DR/ACW0005 trial, taken 3‐5 hours after dosing and at the trough. For each plot, shaded areas represent 95% PI of the median (pink) and 95% PIs of the 2.5th and 97.5th percentile of simulated data (blue). Red lines indicate the median (solid) and 2.5th and 97.5th percentile (dotted) of the observed data. AD, Alzheimer disease; BID, twice daily; MAD, multiple‐ascending‐dose; QD, once daily; PI, prediction interval; PK, pharmacokinetic; VPC, visual predictive check.

The final PPK model was also evaluated by performing a visual predictive check, the results of which are presented separately for Study ACW0001, Study ACW0002, and Study ACW0003 in Figure 2. The results from the visual predictive check procedure confirm that the PK model provides a good description of the plasma concentration data from all 3 populations: healthy volunteers, healthy older participants, and participants with AD.

### Pharmacodynamics

#### Endocrinology

**Figure 3:**
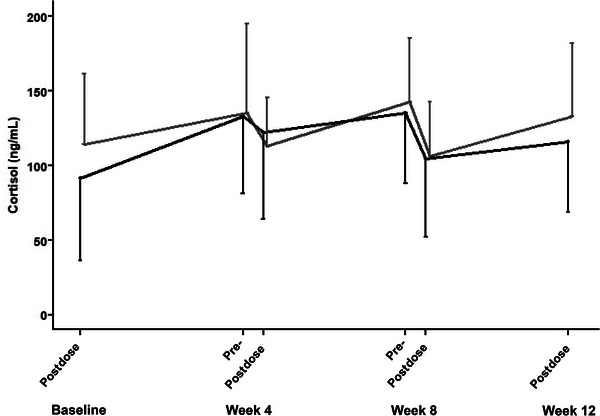
Line plot of mean plasma cortisol level (ng/mL) and standard deviation over time, before dosing, and 3‐5 hours after dosing, for 10 mg emestedastat (black line) and placebo (gray line) in the XanADu trial.

**Figure 4:**
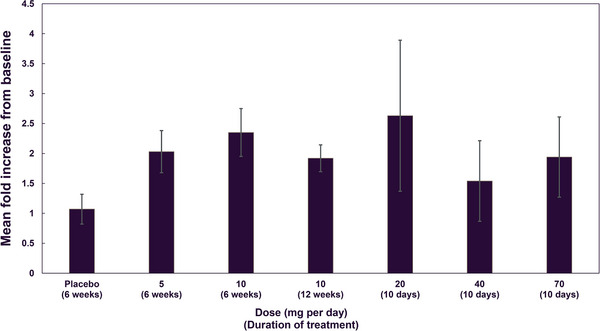
Mean fold increase from baseline in ACTH levels at the end of treatment across multiple‐dose trials. ACTH levels were measured at end of treatment in the MAD (20 mg [n = 6] emestedastat, 40 mg emestedastat [n = 6], and 70 mg emestedastat [n = 6] at 10 days), XanADu (10 mg emestedastat [n = 71] at 12 weeks), and XanaMIA‐DR (placebo [n = 37], 5 mg emestedastat [n = 36], and 10 mg emestedastat [n = 34] at 6 weeks) trials. Error bars represent ± standard error. ACTH, adrenocorticotropic hormone; MAD, multiple‐ascending‐dose.

Webster et al^25^ reported that in the SAD and MAD trials, the endocrine effects of emestedastat administration were no change in plasma cortisol or testosterone, increases in ACTH (to about double baseline), and small rises in DHEAS and androstenedione. These findings were confirmed in the larger and longer XanADu trial. Predose and postdose cortisol (3‐5 hours after dosing) at weeks 4, 8, and 12 are shown in Figure 3 and are within the normal range. There were no differences in plasma cortisol between treatments in change from baseline (*P* = .216). Comparing data across trials, ACTH approximately doubled from baseline without a dose‐response relationship (Figure 4).

### Effects on Cognition

The XanaHES (n = 42) and XanaMIA‐DR (n = 107) trials examined the effect of emestedastat on cognition in healthy older people over 12 and 6 weeks, respectively, using computerized cognitive testing.

In XanaHES, emestedastat‐treated participants displayed better cognitive performance over 12 weeks, with least squares mean differences of −0.04 U on the detection test (90% CI, 0.093‐0.015 U; Cohen's d = 0.49), −0.03 U on the identification test (90% CI, −0.065 to 0.017 U; Cohen's d = 0.65), and −0.06 U on the One Back Test (90% CI, −0.102 to −0.018 U; Cohen's d = 0.98) compared to placebo. The small placebo group of 12 participants displayed an unexpected decrease in performance over 12 weeks, inconsistent with placebo data from the larger cohort in the XanaMIA‐DR trial. Both the emestedastat and placebo groups improved over 12 weeks on the One Card Learning Test, Continuous Paired Associate Learning Test, and the Continuous Paired Associate Learning Test‐Delayed Recall, with no difference between groups (data not shown).

In XanaMIA‐DR, all treatment groups, including placebo, had improved performance on the Detection Test, Identification Test, and One Back Test from baseline to Week 2. The emestedastat groups (5 mg and 10 mg), however, showed further improvement at Weeks 4 and 6. Notably, there was a statistically significant and clinically meaningful improvement in performance in visual attention as measured by the identification test in the 5‐mg emestedastat treatment group of −0.018 U (95% CI, −0.035 to 0.009 U; Cohen's d = 0.32) compared to placebo. This met the a priori criterion for cognition efficacy in the trial. There was no observed dose‐response over 6 weeks of treatment. Although both groups improved over 6 weeks, there was no evidence of any treatment effect in the tests making up the Memory Composite (data not shown).

**Figure 5:**
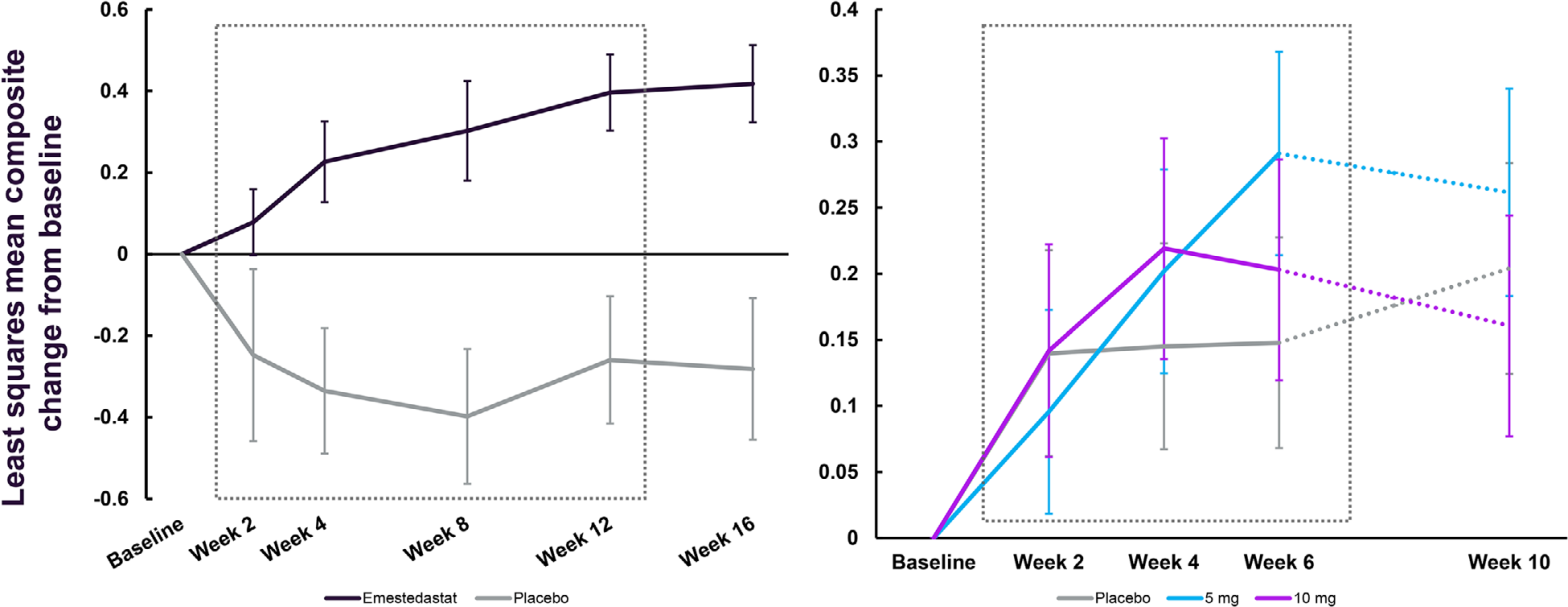
LS mean change and standard error from baseline in scores in the Attention Composite of a CTB in healthy older participants in XanaHES (left) and XanaMIA‐DR (right). Solid lines represent the treatment period, and dotted lines represent the off‐treatment follow‐up period for the trials. For XanaHES, participants received 20 mg emestedastat (black line) or placebo (gray line). For XanaMIA‐DR, participants received 5 mg emestedastat (blue line), 10 mg emestedastat (purple line), or placebo (gray line). CTB, cognitive test battery; LS, least squares.

In both trials, emestedastat displayed a pattern of clinically meaningful improvements in the Attention Composite compared to baseline (Figure 5). In the shorter XanaMIA‐DR trial, the change from baseline in emestedastat‐treated participants (5 or 10 mg) was similar at the end of 6 weeks of treatment compared with those in the XanaHES trial at 8 weeks (20 mg).

Based on these results, a retrospective analysis of the XanADu trial was undertaken. In a prespecified analysis of those participants in the XanADu trial who had plasma biomarker evidence of AD, per elevated plasma pTau181, emestedastat displayed clinical benefit on the Clinical Dementia Ratio—Sum of Boxes compared to placebo (Cohen's d = 0.41). There were also potential benefits observed on the Neuropsychological Test Battery‐Executive domain (Cohen's d = 0.26) but not AD COMposite Scores, ADAS‐Cog14, or Mini‐Mental State Examination (Cohen's d < 0.2).^22^

### Safety and Tolerability

A maximum tolerated dose for emestedastat has not been established in humans. No pattern of symptomatic adverse events has been established, and there have been no serious adverse events attributed to emestedastat in any trial.

## Discussion

Given the time and resources required to test the “cortisol hypothesis” in large clinical trials, evidence of the correct dosing range for such trials is imperative. Peripheral PK/PD methods are of limited utility for the emestedastat program, as the drug acts by inhibiting cerebral tissue cortisol synthesis in particular regions of the brain, while plasma cortisol remains within the normal range in chronic dosing. Thus, plasma PK/PD and even CSF PK may not accurately predict regional target engagement and effects. Functional brain imaging and cognitive testing were pursued to refine the target dosing estimates derived from more traditional PK/PD measures.

Based predominantly on PK in plasma with limited CSF sampling, the initial target dose of 20 mg or more per day appears to have overestimated the likely minimally effective dose evidenced by subsequent brain PET imaging and cognitive testing. The reasons for this may include (1) limited, single‐point CSF sampling in 4 individuals that resulted in an underestimate of the true CSF : plasma ratio; (2) tissue distribution characteristics of the drug to areas of high brain 11β‐HSD1 expression (as better shown functionally on PET); and (3) lack of CNS endocrine PD dose‐response to supplement PK observations. While lack of dose‐response in ACTH levels at doses of 10 mg or more daily pointed to a lower dose range target, it was potentially unreliable as an indicator of brain PD effect, as pituitary regulation is outside the blood‐brain barrier.

The PK was linear over the dose range (10‐70 mg), and early trials found consistent PK/PD between populations of healthy patients and patients with AD, ensuring that the comparability of the safety and activity of doses between the trial populations was sound. Subsequently, the PET trial showed similar regional binding of emestedastat in cognitively normal older participants and participants with AD, further confirming the suitability of combining information across populations.

The linearity of emestedastat plasma PK may also have been a clue to a lower target dose range. A review paper on the PK of 11β‐HSD1 inhibitors as a class demonstrated markedly nonlinear kinetics thought to be due to target‐mediated drug disposition.^28^ The interpretation of these data is that the linear dose range occurs when the peripheral tissue–specific binding, which removes the drug from plasma, is saturated. Hence, it is likely that the peripheral action of emestedastat is maximal at doses of 5 mg or greater due to this being in the linear PK dose range and apparent maximal ACTH increases. This is supported by the near‐maximal suppression of peripheral 11β‐HSD1 activity in the MAD trial at all doses.^25^ The main unknown remaining was the relative exposure levels in brain regions of interest for cognition and the prevention of disease progression.

Trial design or a lack of target engagement may have been factors in the therapeutic failure of ABT‐384, another 11β‐HSD1 inhibitor, in a 12‐week Phase 2 trial in AD,^29^ making the negative result difficult to interpret. Similar to the XanADu trial of emestedastat, the AD population studied was a clinically diagnosed one, without imaging or biomarker confirmation of the AD diagnosis. In addition, the program had no direct demonstration of target activity in the CNS. A report of CNS target engagement via measurements of CSF‐deuterated cortisol may have been due to peripheral enzymatic action.^25^, ^30^

The similar pattern of cognitive benefit on attention and working memory in 2 placebo‐controlled Phase 1b trials was the last piece of the pharmacodynamic puzzle for the emestedastat program and confirms the target dose range indicated in the PET trial. Despite using a population of healthy, cognitively normal, older volunteers, further improvements in 3 of 6 computerized cognition tests were seen with 5, 10, and 20 mg daily. Tests of attention and working memory were improved, whereas tests of episodic memory and executive function were not. Computerized cognitive testing has considerable appeal in PD assessment for cognitive‐enhancing drugs in early clinical trials. The test batteries can be easily incorporated into trials with other objectives and assessments; they have minimal need for operator training, there are minimal learning or placebo effects after suitable training, and there is a considerable database of normal ranges and clinical effect sizes with various drug classes.^31^, ^32^

One limitation of the generalizability of these methods to optimize emestedastat dosing is that other cognitively enhancing drugs such as donepezil may not reliably show benefits in cognitively normal volunteers.^33^, ^34^ In contrast, carbenoxolone, a nonselective 11β‐HSD inhibitor, improved the verbal fluency test, a measure of executive function, in a small, double‐blind, crossover trial in 10 healthy older (55‐75 years) men of 4 weeks’ duration. In another part of that trial in 12 patients with type 2 diabetes mellitus, there was improved verbal memory in the Rey Auditory Verbal Learning Test, and there were nonsignificant improvements in other cognitive tests.^15^ Carbenoxolone, however, is not suitable as chronic therapy for CNS indications, as it also inhibits 11β‐HSD2 in the kidney, leading to mineralocorticoid effects, with salt retention and hypertension side effects (11β‐HSD2 is absent from the forebrain, including from cognitive circuitry). However, the demonstration of pro‐cognitive effects of carbenoxolone in patients without cognitive impairment in a small, short‐term trial provides additional support to the likely utility of cognitive function and testing with emestedastat.

The endocrinology findings with emestedastat are generally consistent with other 11β‐HSD1 inhibitors such as ABT‐384, J2H‐1702, and BI‐187004.^35^, ^36^, ^37^ Typical findings include a rise in ACTH, DHEAS, and androstenedione and the lack of changes at steady state in plasma testosterone and serum cortisol. An approximately 2‐fold rise in ACTH is a biomarker for inhibition of peripheral (liver and splanchnic bed) 11β‐HSD1 seen with all peripherally targeted drugs studied to date, as well as in preclinical studies with mouse 11β‐HSD1 gene (*Hsd11b1*) knockouts.^38^ This incremental rise in plasma ACTH may be due to the lower level of cortisol synthesis in peripheral tissues such as liver and adipose tissues, as these contribute to plasma cortisol levels. In the absence of such contribution, ACTH synthesis is increased to maintain normal plasma cortisol levels. The increases in DHEAS and androstenedione are consequent to this rise in ACTH. However, as this is not the major pathway of testosterone synthesis, testosterone levels are unchanged. As 11β‐HSD1 is not the enzyme responsible for adrenal synthesis of cortisol, changes in serum cortisol would not be expected, and this was confirmed in the XanADu trial.

We have demonstrated in this series of clinical trials that in a condition such as AD, where the failure rate of new therapies is high, confirmation of activity and optimal dose selection of emestedastat can be achieved with moderate resource usage using a multiple biomarker approach, including brain PET imaging and cognitive testing. The use of a newly available plasma biomarker, elevated pTau181, unmasked potential efficacy in AD and allowed simulation of a Phase 2b trial based on prior Phase 2a clinical data. The combined data set provides sufficient confidence to proceed to further development at a dose of 10 mg daily. While no one trial was pivotal, the combination of data from multiple trials supports the likely clinical activity of emestedastat in AD and potentially other diseases. This will need to be confirmed by further clinical efficacy trials.

## Conflicts of Interest

Jack Taylor and Dana Hilt are employees of Actinogen Medical. Paul Rolan, Mark Jaros, and John Harrison are paid consultants to Actinogen Medical.

## Funding

All trials were funded by Actinogen Medical.

## Supporting information



Supporting Information
