# Supplementary material for: Clinical Pharmacology and Approach to Dose Selection of Emestedastat, a Novel Tissue Cortisol Synthesis Inhibitor for the Treatment of Central Nervous System Disease
Source: Clin Pharmacol Drug Dev. 2025 Jan 2;14(2):105–15. doi: 10.1002/cpdd.1496 (PMC11788964; doi:10.1002/cpdd.1496)
Supplement: Supplementary file 1 — Supporting Information [file CPDD-14-105-s001.pdf]

Supplemental Digital Content

Methods

Completed Clinical Trials

Table S1 Details of clinical trial sites and ethics committees for clinical trials with emestedastat.

Clinical Trial Sites		
**Trial Identifier (NCT Reference)**	**Name of Site**	**Location of Site**
RD 656/25368	Simbec Research Ltd	Merthyr Tydfil, Wales, UK
ACW0001	Linear Clinical Research Ltd	Nedlands, Western Australia, Australia
XanaHES ACW0003 (NCT03830762)[a]	Linear Clinical Research Ltd	Nedlands, Western Australia, Australia
ACW0004	Austin Health	Heidelberg, Victoria, Australia
XanADu ACW0002 (NCT02727699)[b]	Central Coast Neurosciences Research (# 101)	Tumbi Umbi, East Gosford, and Erina, New South Wales, Australia
	Austin Health (#102)	Heidelberg, Victoria, Australia
	Australian Alzheimer's Research Foundation (#103)	Nedlands, Western Australia, Australia
	St. Vincent's Hospital - Centre for Applied Medical Research (#104)	Darlinghurst, New South Wales, Australia
	KaRa Institute of Neurological Diseases Pty Ltd (#105)	Macquarie Park, New South Wales, Australia
	Royal Melbourne Hospital (#106)	Parkville, Victoria, Australia
	Richmond Behavioral Associates (#201)	Staten Island, New York, USA
	The Clinical Trial Center (#203)	Jenkintown, Pennsylvania, USA
	Atlanta Center for Medical Research (#204)	Atlanta, Georgia, USA
	Bioclinica Research, Orlando (#207)	Orlando, Florida, USA
	PMG Research of Rocky Mount, LLC/ Boice-Willis Clinic (#215)	Rocky Mount, North Carolina, USA
	The Neurocognitive Institute / Exodon (#216)	Mount Arlington, New Jersey, USA
	Northern Clinical Research (#217)	Sacramento, California, USA
	IMIC, Inc. (#218)	Palmetto Bay, Florida, USA
	PCND Neuroscience Research Institute / San Diego Pain Consultants, Inc. (#219)	Poway, California, USA
	National Research Institute (#220)	Los Angeles, California, USA

**Clinical Trial Sites**		
	Neurostudies Net, LLC (#222)	Decatur, Georgia, USA
	Tucson Neuroscience Research, LLC (#223)	Tucson, Arizona, USA
	The Neurology Research Group, LLC (#224)	Miami, Florida, USA
	Research Alliance, Inc. (#225)	Clearwater, Florida, USA
	Pacific Research Network, Inc. (#226)	San Diego, California, USA
	Belfast Health And Social Care Trust, Queen's University Belfast (#301)	Belfast, Northern Ireland, UK
	Centre for Clinical Brain Sciences, Centre for Dementia Prevention - The University of Edinburgh (#302)	Edinburgh, Scotland, UK
	The Research Institute for the Care of Older People Centre - Royal United Hospital (#305)	Bath, England, UK
	West London Mental Health Trust - Lakeside Mental Health Unit (#308)	Isleworth, England, UK
	Manchester Mental Health & Social Care Trust - Dementia Research Office - Park House North Manchester General Hospital (#312)	Manchester, England, UK
	St. Pancras Clinical Research (#313)	London, England, UK
XanADu biomarker extension ACW0002A	The following sites from trial ACW0002: 101, 102, 103, 105, 203, 204, 218, 219, 224, 301, 302, 305, 308, 312, 313	As above
XanaMIA-DR ACW0005 (NCT04983368)[c]	Paratus Clinical Pty Ltd (#001)	Central Coast, New South Wales, Australia
	Paratus Clinical Pty Ltd (#002)	Canberra, Australian Capital Territory, Australia
	Paratus Clinical Pty Ltd (#003)	Western Sydney, New South Wales, Australia
	Paratus Clinical Pty Ltd (#004)	Brisbane, Queensland, Australia
	University of Sunshine Coast (#006)	Sippy Downs, Queensland, Australia
**Ethics Committee Details**		
**Trial Identifier (NCT Reference)**	**Name of Ethics Committee**	**Location of Ethics Committee**
RD 656/25368	South East Wales Research Ethics Committee – Panel D	Cardiff, Wales, UK
ACW0001	Bellberry Ltd Human Research Ethics Committee	Eastwood, South Australia, Australia
XanaHES ACW0003 (NCT03830762)[a]	Bellberry Ltd Human Research Ethics Committee	Eastwood, South Australia, Australia

Clinical Trial Sites		
ACW0004	Austin Health Human Research Ethics Committee	Heidelberg, Victoria, Australia
XanADu<br>ACW0002	Bellberry Ltd Human Research Ethics Committee	Eastwood, South Australia, Australia
(NCT02727699)[b]	Austin Health Human Research Ethics Committee	Heidelberg, Victoria, Australia
	Copernicus Group IRB	Durham, North Carolina, USA<br>Cary, North Carolina, USA
	BioMed IRB	San Diego, California, USA
	Scotland A Research Ethics Committee	Edinburgh, Scotland, UK
XanADu biomarker extension<br>ACW0002A	As per trial ACW0002	As per trial ACW0002
XanaMIA-DR<br>ACW0005<br>(NCT04983368)[c]	Bellberry Ltd Human Research Ethics Committee	Eastwood, South Australia, Australia

[a] https://clinicaltrials.gov/study/NCT03830762
[b] https://clinicaltrials.gov/study/NCT02727699
[c] https://clinicaltrials.gov/study/NCT04983368

Bioanalysis

UE2343/emestedastat in plasma and CSF in the SAD and MAD trials were assayed by LC-MS/MS using liquid extraction with 0.1% formic acid with evaporation to dryness and reconstitution of 85% 0.1% formic acid: 15% methanol using phenacetin (100µL; 0.5 µg/ml) as an internal standard. Chromatography was performed on a C18 column with gradient elution using mobile phases of 0.1% formic acid in deionised water and 0.1% formic acid in acetonitrile. MS/MS for detection was used in Multiple Reaction Monitoring mode using *m/z* transition for UE2343 of 382-78.1 and 108.2-110.1 for phenacetin. The assay was validated over the range 5 – 500 ng/mL. For trials ACW0002, ACW0003, and ACW0005, the assay was modified by preparing the samples by protein precipitation and using phenacetin-d_3 as the internal standard. MS/MS for detection was used in Multiple Reaction Monitoring mode using *m/z* transition for UE2343 of 382.3-177.2 and 183.2-111.1 for phenacetin-d_3. The validation range was 10 - 5000 ng/mL.

A range of bioassays were used for determination of cortisol, testosterone, androstenedione, DHEAS, and ACTH in the clinical trials, as summarized in Table S2.

Table S2 Details of analytical methods used for determination of pharmacodynamics hormones

Analyte	Trial	Method of Analysis
ACTH	SAD/ RD 656/25368	Chemiluminescence (Siemens Immulite 1000 or 2000)
	MAD/ACW0001	Chemiluminescence (Siemens Immulite 1000 or 2000)
	XanaDu/ACW0002	ELISA
	XanaHES/ACW0003	ELISA
	XanaMIA-DR/ACW0005	Chemiluminescence (Siemens Immulite 1000 or 2000)

Cortisol	SAD/ RD 656/25368	Immunoassay (Roche Cobas)
	MAD/ACW0001	Immunoassay (Roche Cobas)
	XanaDu/ACW0002	LC-MS/MS
	XanaHES/ACW0003	LC-MS/MS
Testosterone	SAD/ RD 656/25368	Immunoassay (Roche Cobas)
	MAD/ACW0001	Immunoassay (Roche Cobas)
	XanADu/ACW0002	LC-MS/MS
	XanaHES/ACW0003	LC-MS/MS
Androstenedione	SAD/ RD 656/25368	Immunohistochemistry (ICN ImmuChem Double Antibody androstenedione)
	MAD/ACW0001	Immunohistochemistry (ICN ImmuChem Double Antibody androstenedione)
	XanaDu/ACW0002	LC-MS/MS
	XanaHES/ACW0003	LC-MS/MS
DHEAS	SAD/ RD 656/25368	Chemiluminescence (Siemens Immulite 1000 or 2000)
	MAD/ACW0001	Chemiluminescence (Siemens Immulite 1000 or 2000)
	XanaDu/ACW0002	LC-MS/MS
	XanaHES/ACW0003	LC-MS/MS

For the LC-MS/MS assays of cortisol, testosterone, androstenedione and DHEAS, plasma samples were prepared by acetonitrile protein precipitation, filtration, evaporated to dryness and then dissolved with 70% water:30% ethanol. Samples for DHEAS analysis were diluted 5-fold with water. 2,3,4 [13C3]-testosterone (13C3-T) was used as internal standard for testosterone, 2,3,4 [13C3]-androstenedione (13C3-A4) was used as internal standard for androstenedione, 2,2,3,4,4 [2H5]-dehydroepiandrosterone sulphate (d5-DHEA-S) was used as internal standard for dehydroepiandrosterone sulphate and 9,11,12,12 [2H4]-cortisol (d4-F) was used as internal standard for cortisol. Chromatography was performed on a biphenyl column using gradient elution with mobile phases of 0.05 mM ammonium fluoride in water and 0.05 mM ammonium fluoride in methanol. Detection by Multiple Reaction Monitoring was using the *m/z* values in Table S3.

Other assays were performed in accordance with the manufacturer's instructions.

Table S3 *m/z* values used for Multiple Reaction Monitoring in LC-MS/MS assays

ID	Q1 *m/z*	Q3 *m/z*	Polarity	Expected RT (mins)
Cortisol	363.1	121.1	+	5.45
Testosterone	289.1	97.0	+	13.6
Androstenedione (A4)	287.0	97.0	+	16.0
DHEA-S	367.1	96.9	-	2.76
d4-Cortisol (d4F)	367.3	121.1	+	5.39
13C3-Testosterone (13C3-T)	292.1	100.0	+	13.6
13C3-Androstenedione (13C3A4)	290.2	100.1	+	16.0
[2H5]-dehydroepiandrosterone sulphate (d5-DHEA-S)	372.1	97.9	-	2.74

Figure Legend: Supplemental Digital Content

Figure S1

Composite ^{11}C-TARACT images at baseline and with increasing emestedastat dosage. Composite images (n=8 participants in each dose group) of standardized uptake value taken at 40 to 60 minutes post injection of tracer before (top) and after (bottom) 7 days of emestedastat treatment with 5 mg, 10 mg, 20 mg, and 30 mg once daily in the morning, shown in ascending dose order from left to right. Figure reproduced with permission[21].

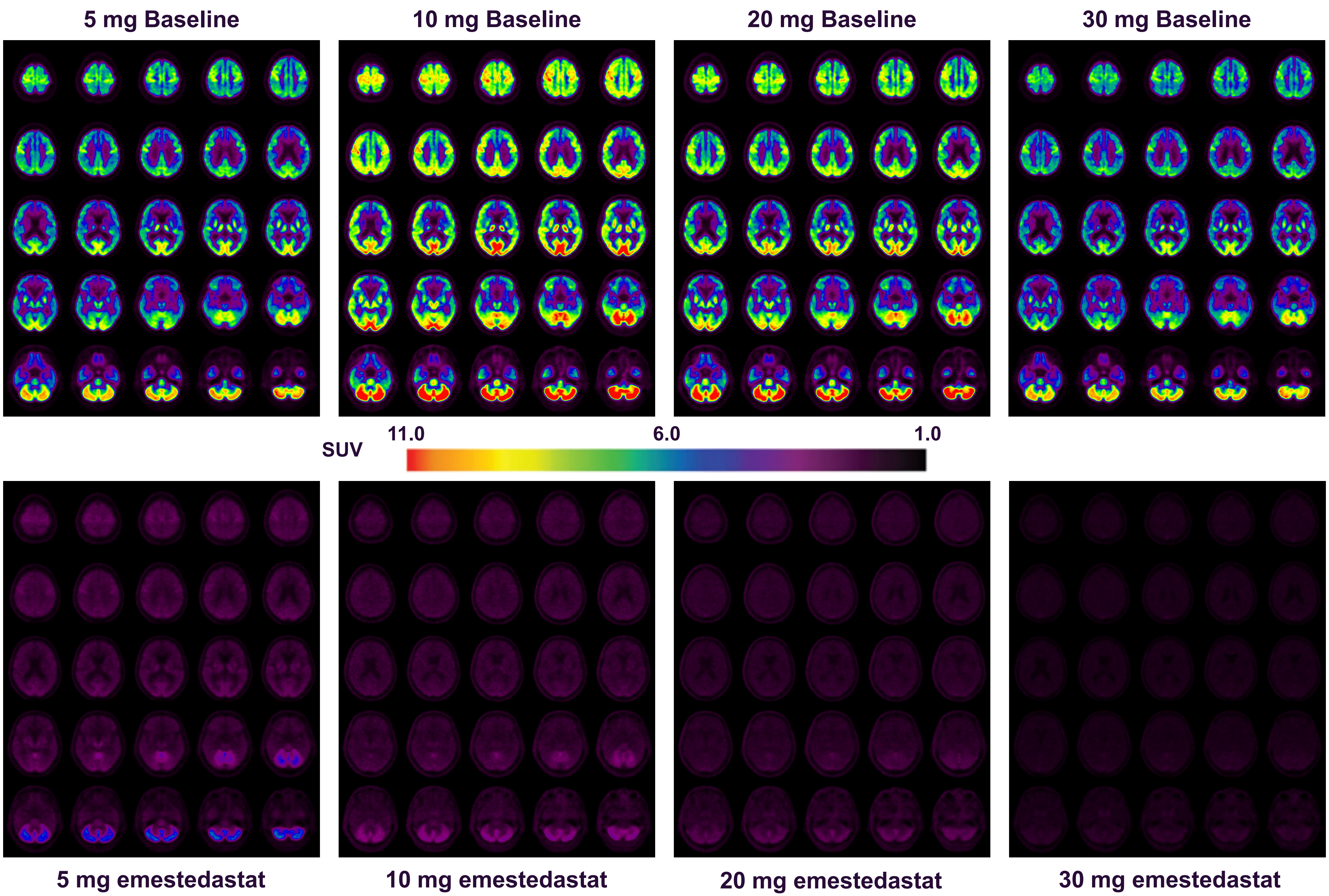

5 mg Baseline
10 mg Baseline
20 mg Baseline
30 mg Baseline
SUV
11.0
6.0
1.0
5 mg emestedastat
10 mg emestedastat
20 mg emestedastat
30 mg emestedastat
